# Protein, dietary fiber, minerals, antioxidant pigments and phytochemicals, and antioxidant activity in selected red morph *Amaranthus* leafy vegetable

**DOI:** 10.1371/journal.pone.0222517

**Published:** 2019-12-12

**Authors:** Umakanta Sarker, Shinya Oba

**Affiliations:** 1 Department of Genetics and Plant Breeding, Faculty of Agriculture, Bangabandhu Sheikh Mujibur Rahman Agricultural University, Gazipur, Bangladesh; 2 Laboratory of Field Science, Faculty of Applied Biological Sciences, Gifu University, Yanagido, Gifu, Japan; Higher Institute of Applied Sciences and Technology of Gabes University of Gabes, TUNISIA

## Abstract

Amaranth has two morphological types (morphs), one is red and another is green morph. Red morph amaranth is a marvelous source of nutrients, antioxidant pigments, minerals, and phytochemicals compared to green morph amaranth. For this purpose, we selected 25 red morph genotypes to evaluate in terms of proximate, minerals, antioxidant pigments and phytochemicals and antioxidant activity in RCBD design in three replicates. The leaves of red morph amaranth are an excellent source of dietary fiber, carbohydrates, moisture, and protein. We found remarkable potassium, calcium, magnesium (24.96, 10.13, 30.01 mg g^-1^), iron, manganese, copper, zinc (1089.19, 243.59, 25.77, 986.61 μg g^-1^), chlorophyll *a*, chlorophyll *b* (31.79, 16.05 mg 100 g^-1^), β-cyanins, total flavonoids (102.10 RE μg g^-1^ DW), β-xanthins, betalains (33.30, 33.09, 66.40 μg 100 g^-1^), carotenoids, total phenolics (172.23 GAE μg g^-1^ DW), β-carotene (1225.94, 1043.18 μg g^-1^), vitamin C (955.19 μg g^-1^), and antioxidant activity (DPPH and ABTS^+^) (19.97 and 39.09 TEAC μg g^-1^ DW) in the red morph amaranth leaves. We can select the genotype RA5, RA8, RA18, RA22, and RA25 as antioxidant-enriched red morph amaranth. It revealed that amaranth β-cyanins, phenolics, betalains, flavonoids, β-xanthins, carotenoids, vitamin C, and β-carotene had strong antioxidant activity. These phytochemicals contributed significantly in the antioxidant potentials of red morphs amaranth. Red morph amaranth could be a potential source of nutrients, antioxidant pigments, minerals, and phytochemicals as these compounds scavenged ROS and served as potential antioxidants in our daily diet to attaining nutritional and antioxidant sufficiency.

## Introduction

The acceptability of foods largely depends on the color of the food products. Recently, the demand for natural pigments such as carotenoids, β-xanthins, β-cyanins, anthocyanin, betalains, and chlorophylls have increased the interest in consumers in the safety, nutritional, and aesthetic aspects of food. A few families in the order Caryophyllales have water-soluble natural pigments like β-cyanins, β-xanthins, and betalains. *Amaranthus* (red amaranth) is a unique source of betalains, β-xanthins, β-cyanins that have potential free radical detoxifying ability [1]. Red to purple colored betalains are β-cyanins and yellow colored betalains are β-xanthins [2]. Similarly, α-carotene, xanthophyll, and beta-carotene are different carotenoids pigments. Among edible vegetables, red beet and amaranth have natural pigments, like betalains, β-cyanins, β-xanthins. Red morph *Amaranthus* is a marvelous source of color pigments like β-cyanins, β-xanthins, betalains, anthocyanin, amaranthine, carotenoids, and chlorophylls. These pigments detoxify free radicals in the human body and act as potent antioxidants [3] and have a significant contribution to human health. The anti-inflammatory property of the active ingredients of carotenoids, betalains, β-cyanins, and β-xanthins protect against lung and skin cancers and cardiovascular disease. For this reason, these natural pigments are widely used as an additive for cosmetic products, drugs, and food [4].

Vegetable amaranth is a C_4_ leafy vegetable. It is a marvelous source of proximate, minerals, phytopigments, bioactive compounds that had pronounced significance as a food natural antioxidants and ROS scavenger [5–10]. It is inexpensive and abundant sources of protein, dietary fiber, pigments, minerals and antioxidant phytochemicals like flavonoids, β-carotene, phenolics, and vitamin C. Amaranth protein are enriched with nutritionally important amino acids such as lysine and methionine [11–14]. It is also tolerant to abiotic stresses like drought and salinity [15–20]. Amaranth has two morphological types (morph), one is red and another is green morph [21]. Red morph amaranth is an abundant source of pigments as well as minerals, proximate, bioactive phytochemicals, and antioxidants. There are a lot of red morph amaranth germplasms available in Bangladesh, Asia, Africa and South America with great variability and phenotypic plasticity [22] that have multipurpose uses. In Bangladesh including south-east Asia, Africa, and South America, red morph amaranth leaves are very popular as a vegetable. Its nutritional value, taste, and attractive leaf color make it popular in the rest of the continent and elsewhere. In Bangladesh, red morph amaranth is grown year-round and it can be grown in the hot summer, a gap period of foliage vegetables [11–12].

Recently, researchers and consumers have shown interest in natural antioxidants in red morph vegetables. Red morph amaranth has abundant flavonoids, pigments, β-carotene, phenolics, and vitamin C [13, 23]. These natural antioxidants protect cancer, emphysema, cardiovascular diseases, atherosclerosis, diabetes, retinopathy, osteoporesis, neurodegenerative diseases, arthritis, cataracts, inflammation, and prevent aging [23–25].

Although red morph amaranth is a cheap and abundant source of minerals, pigments, dietary fiber, phytochemicals, protein, and antioxidant activity. There is a scarce of information in red morph *Amaranthus* leafy vegetable. To our knowledge, there is a lack of information on proximate and mineral compositions, pigments, phytochemicals, and antioxidant activity in a huge number of diversified red morph amaranth germplasms available in Bangladesh and elsewhere. Therefore, to fill these gaps, the present investigation was undertaken to evaluate proximate and mineral compositions, antioxidant pigments, phytochemicals, and antioxidant activity and their variability in 25 red morph amaranth genotypes.

## Materials and methods

### Experiment materials, design, layout, and cultural practices

Twenty-five selected genotypes of red amaranth from our earlier collected 120 germplasm were grown in open field of Bangabandhu Sheikh Mujibur Rahman Agricultural University in a randomized complete block design (RCBD) with three replications. The unit plot size of each genotype was 1 square meter. The spacing of each red amaranth genotype was 20 cm distance from row to row and 5 cm distance from the plant to plant. Recommended fertilizer, compost doses, and appropriate cultural practices were maintained. Thinning was done to maintain appropriate spacing between plants of a row. As a necessity, weeding and hoeing were done to remove the weeds. To maintain the normal growth of the crop proper irrigations were provided. At 30 days after sowing of seed, leaf samples were collected.

### Chemicals

Solvent: acetone and methanol. Reagents: H_2_SO_4_, HNO_3_, HClO_3,_ NaOH, dithiothreitol (DTT), caesium chloride, ascorbic acid, standard compounds of pure Trolox (6-hydroxy-2, 5, 7, 8-tetramethyl-chroman-2-carboxylic acid), gallic acid, rutin, folin-ciocalteu reagent, DPPH (2, 2-diphenyl1-picrylhydrazyl), ABTS^+^, aluminium chloride hexahydrate, sodium carbonate, potassium acetate, and potassium persulfate. All solvents and reagents were bought from Merck (Germany) and Kanto Chemical Co. Inc. (Tokyo, Japan).

### Estimation of proximate composition

AOAC method was followed [26] to estimate the ash, moisture, crude fat, fiber, crude protein contents, and gross energy. Micro-Kjeldahl method was followed to determine crude protein multiplying nitrogen by 6.25 (AOAC method 976.05). The sum of crude protein, moisture, crude fat, and ash percentage was subtracted from 100 to estimate carbohydrate (g kg^-1^ FW).

### Determination of mineral composition

Leaves of red amaranth were dried at 70°C in an oven for 24 hours. Dried leaves were grounded finely in a mill. Nitric-perchloric acid digestion method [26] was followed to determine calcium, potassium, magnesium, iron, manganese, copper, and zinc from powdered leaves. For this digestion, in the presence of carborundum beads 40 ml HClO_4_ (70%), 400 ml HNO_3_ (65%), and 10 ml H_2_SO_4_ (96%) were added to 0.5 g dried leaf sample. After digestion, the solution was appropriately diluted in triplicate for measuring phosphorus following ascorbic acid method. Addition of ascorbic acid and antimony to the yellow-colored complex solution converted to a blue-colored phosphomolybdenum complex. Sarker and Oba [26] method was followed to read the absorbance by atomic absorption spectrophotometry (AAS) (Hitachi, Tokyo, Japan) at a wavelength of 285.2 nm (magnesium), 76 6.5 nm (potassium), 248.3 nm (iron), 422.7 nm (calcium), 279.5 nm (manganese), 213.9 nm (zinc), 324.8 nm (copper).

### Determination of chlorophylls and carotenoids

The leaves of red amaranth were extracted in 80% acetone to estimate chlorophyll *ab*, chlorophyll *b*, total carotenoids, and chlorophyll *a* according to the method of Sarker and Oba [26]. A spectrophotometer (Hitachi, U-1800, Tokyo, Japan) was used to read the absorbance at 663 nm for chlorophyll *a*, 646 nm for chlorophyll *b*, and 470 nm for total carotenoids, respectively. Data were calculated as mg chlorophyll per 100 g fresh weight (FW) and μg total carotenoids per g FW.

### Determination of β-cyanins and β-xanthins content

The leaves of red amaranth were extracted in 80% methyl alcohol having 50 mM ascorbate to measure β-cyanins and β-xanthins according to the method of Sarker and Oba [26]. A spectrophotometer (Hitachi, U-1800, Tokyo, Japan) was used to measure the absorbance at 540 nm for β-cyanins and 475 nm for β-xanthins, respectively. The results were expressed as microgram betanin equivalent per 100 gram FW for β-cyanins and micrograms indicaxanthin equivalent per 100 gram FW for β-xanthins.

### Estimation of β-carotene

Method of Sarker and Oba [26] was followed to extract and determine β-carotene content. In a mortar and pestle, 10 ml of 80% acetone was added in 500 mg of fresh leaf sample and ground thoroughly. The extract was centrifuged at 10,000 rpm for 3–4 min. The final volume was brought up to 20 ml after removing the supernatant in a volumetric flask. A spectrophotometer (Hitachi, U-1800, Tokyo, Japan) was used to take the absorbance at 510 nm and 480 nm. Data were expressed as μg β-carotene per g fresh weight (FW).

The following formula was used to measure the β-carotene content:

β-carotene = 7.6 (Abs. at 480) - 1.49 (Abs. at 510) × Final volume/ (1000 × fresh weight of leaf taken)

### Estimation of Vitamin C

The fresh red amaranth leaves were used to measure ascorbate (AsA) and dehydroascorbic acid (DHA) acid through a spectrophotometer. For pre-incubation of the sample and reduction of DHA into AsA Dithiothreitol (DTT) was used. AsA reduced Fe_3_^+^ to Fe_2_^+^ and estimation of AsA was made by the spectrophotometric (Hitachi, U-1800, Tokyo, Japan) measuring Fe_2_^+^ complexes with 2, 2-dipyridyl [26]. Finally, the absorbance of the sample solution was read. Data were recorded as μg vitamin C per g fresh weight (FW).

### Sample extraction for TPC, TFC and TAC analysis

30 DAS red amaranth leaves were harvested. For chemical analysis, the leaves were dried in the air in a shade. 40 ml of 90% aqueous methanol was used to extract 1 g of grounded dried leaves from each cultivar in a bottle (100 ml) capped tightly. A shaking water bath (Thomastant T-N22S, Thomas Kagaku Co. Ltd., Japan) was used to the extract for 1 h. The extract was filtered for determination of polyphenols, flavonoids, total antioxidant capacity.

### Determination of polyphenols

Method of Sarker and Oba [27] was followed to estimate the total phenolic content of red amaranth using the folin-ciocalteu reagent with gallic acid as a standard phenolic compound. Folin-ciocalteu reagent was previously diluted 1:4, reagent: distilled water. In a test tube, 1 ml of diluted folin-ciocalteu was added to 50 μl extract solution and then mixed thoroughly for 3 min. 1 ml of Na_2_CO_3_ (10%) was added to the tube and stand for 1 h in the dark. A Hitachi U1800 spectrophotometer (Hitachi, Tokyo, Japan) was used to read the absorbance at 760 nm. A standard gallic acid graph was made to determine the concentration of phenolics in the extracts. The results are expressed as μg gallic acid equivalent (GAE) g^-1^ DW.

### Determination of flavonoids

The AlCl_3_ colorimetric method [28, 29] was used to estimate the total flavonoid content of red amaranth extract. In a test tube, 1.5 ml of methanol was added to 0.1 ml of 10% aluminum chloride, 0.1 ml of 1 M potassium acetate, 2.8 ml of distilled water and 500 μl of leaf extract for 30 min at room temperature. A Hitachi U1800 spectrophotometer (Hitachi, Tokyo, Japan) was used to take the absorbance of the reaction mixture at 415 nm. TFC is expressed as μg rutin equivalent (RE) g^-1^ dry weight (DW) using rutin as the standard compound.

### Antioxidant capacity (TAC)

Diphenyl-picrylhydrazyl (DPPH) radical degradation method [30] was used to estimate the antioxidant activity. In a test tube, 1 ml of 250 μM DPPH solution was added to 10 μl of leaf extract solution (in triplicate) and 4 ml of distilled water and allowed to stand for 30 min in the dark. A Hitachi U1800 spectrophotometer (Hitachi, Tokyo, Japan) was used to read the absorbance at 517 nm. Method of Sarker and Oba [31] was followed for ABTS^+^ assay. 7.4 mM ABTS^+^ solution and 2.6 mM potassium persulfate were used in the stock solutions. The two stock solutions were mixed in equal quantities and allowing them to react for 12 h at room temperature in the dark for preparation of the working solution. 2850 μl of ABTS^+^ solution (1 ml ABTS^+^ solution mixed with 60 ml methanol) was mixed with 150 μl sample of leaf extract and allowed to react for 2 h in the dark. A Hitachi U1800 spectrophotometer (Hitachi, Tokyo, Japan) was used to read the absorbance against methanol at 734 nm. The percent of inhibition of DPPH and ABTS^+^ relative to the control were used to determine antioxidant activity using the following equation:

Antioxidant activity (%) = (Abs. blank- Abs. sample/Abs. blank) × 100

Where, Abs. blank is the absorbance of the control reaction [10 μl methanol for TAC (DPPH), 150 μl methanol for TAC (ABTS^+^) instead of leaf extract] and Abs. sample is the absorbance of the test compound. Trolox was used as the reference standard, and the results were expressed as μg Trolox equivalent g^-1^ DW.

### Statistical analysis

At first, sample data of each trait were averaged replication-wise. The mean data of three replications for all traits were also statistically analyzed by ANOVA using Statistix 8 software, and the means were compared by the Tukey’s HSD test at 1% level of probability. The results were reported as the average of three replications ± SD.

## Results

The analysis of variance demonstrated that all the traits significantly varied between the different studied genotypes (Tables 1, 2, 3 and 4). Proximate and mineral compositions, antioxidant leaf pigments, vitamins, TAC (DPPH), TFC, TPC, and TAC (ABTS^+^) of the 25 tested red morph amaranth genotypes are presented in Tables 1, 2, 3 and 4.

**Table 1:** Proximate compositions (g kg^-1^ fresh weight) and dietary fiber (μg g^-1^ FW) of 25 red morph amaranth genotypes.

Genotypes	Moisture (g kg^-1^)	Protein (g kg^-1^)	Fat (g kg^-1^)	Carbohydrates (g kg^-1^)	Energy (Kcal)	Ash (g kg^-1^)	Dietary fiber (μg g^-1^ FW)
RA1	846.52 ± 2.37i	24.37 ± 0.72m	2.14 ± 0.02q	90.27 ± 0.99b	47.58 ± 2.12d	36.25 ± 1.12f	85.62 ± 2.15c
RA2	865.75 ± 1.97e	15.63 ± 1.23o	3.32 ± 0.04h	81.68 ± 1.21e	37.42 ± 1.82k	34.14 ± 0.88h	91.66 ± 1.25a
RA3	814.64 ± 2.12n	62.26 ± 1.28a	1.85 ± 0.06r	64.94 ± 2.15j	55.33 ± 1.59a	56.55 ± 1.01a	67.72 ± 1.16h
RA4	870.45 ± 3.21d	36.53 ± 0.85g	2.86 ± 0.05k	61.16 ± 1.87k	41.06 ± 2.25i	29.65 ± 1.11j	88.72 ± 0.87b
RA5	827.48 ± 2.46l	42.64 ± 1.12f	1.63 ± 0.03s	75.28 ± 1.28g	45.93 ± 2.74f	53.52 ± 1.24b	77.75 ± 1.23e
RA6	854.60 ± 1.87h	11.38 ± 1.25q	2.55 ± 0.02n	97.51 ± 1.25a	46.32 ± 3.12e	34.58 ± 1.37h	73.41 ± 1.27g
RA7	880.54 ± 3.09b	21.55 ± 1.21n	4.35 ± 0.01a	61.43 ± 2.16k	35.78 ± 1.99l	32.72 ± 1.09i	67.16 ± 1,45i
RA8	822.56 ± 3.22m	51.86 ± 1.43d	2.41 ± 0.02o	71.26 ± 1.27i	53.38 ± 1.17b	54.36 ± 1.17b	82.75 ± 1.38d
RA9	864.35 ± 2.37f	42.49 ± 1.75f	4.23 ± 0.01b	58.11 ± 2.01l	41.02 ± 2.26i	31.22 ± 1.32i	85.74 ± 1.65c
RA10	856.46 ± 3.65g	35.37 ± 2.08h	2.74 ± 0.03l	82.66 ± 2.25e	46.23 ± 3.06f	22.88 ± 1.46k	83.85 ± 1.28d
RA11	884.73 ± 4.24a	55.65 ± 0.88b	3.58 ± 0.02f	22.15 ± 1.87m	35.91 ± 2,64l	34.65 ± 1.15g	74.54 ± 1.18g
RA12	857.47 ± 4.07g	25.72 ± 1.32k	3.66 ± 0.01e	73.84 ± 1.26h	42.64 ± 1.88h	39.28 ± 1.24e	62.42 ± 0.86j
RA13	878.22 ± 3.86c	31.87 ± 1.19j	2.42 ± 0.04o	58.82 ± 1.85l	36.05 ± 2.06l	28.84 ± 1.18j	73.83 ± 1.65g
RA14	866.62 ± 4.19e	32.25 ± 0.95j	2.62 ± 0.03m	75.29 ± 1.75g	44.56 ± 2.36g	22.58 ± 1.25k	76.65 ± 2.31f
RA15	821.58 ± 5.32m	53.88 ± 1.07c	2.31 ± 0.03p	71.41 ± 1.79i	52.99 ± 2.23b	51.55 ± 1.18c	78.21 ± 2.64e
RA16	837.49 ± 6.57j	14.77 ± 2.12p	2.68 ± 0.04l	98.54 ± 1.28a	46.61 ± 1.68d	46.26 ± 0.99d	83.56 ± 2.85d
RA17	854.55 ± 5.38h	23.58 ± 1.54m	2.78 ± 0.02l	84.65 ± 1.18d	41.72 ± 2.06h	34.46 ± 1.07h	78.73 ± 1.28e
RA18	814.83 ± 5.12n	54.53 ± 1.62c	2.86 ± 0.01k	77.28 ± 1.29f	56.07 ± 2.34a	51.23 ± 0.87c	77.21 ± 1.23e
RA19	884.54 ± 4.89a	44.98 ± 0.99e	2.58 ± 0.03m	15.48 ±1.36m	26.95 ± 2.91m	52.38 ± 1.12c	68.85 ± 2.16h
RA20	835.25 ± 3.75k	24.82 ± 0.76l	4.21 ± 0.02c	89. 44 ± 1.66b	45.14 ± 1.22g	46.85 ± 0.85d	82.43 ± 2.34d
RA21	869.74 ± 5.25d	24.62 ± 1.26m	3.15 ± 0.03j	69.85 ± 1.25i	36.27 ± 2.15l	33.43 ± 1.04h	87.64 ± 1.65b
RA22	847.68 ± 2.76i	15.65 ± 1.82o	4.14 ± 0.03d	87.12 ± 1.17c	40.15 ± 2.31i	45.57 ± 0.93d	87.82 ± 2.03b
RA23	864.92 ± 4.39f	33.87 ± 1.16i	3.45 ± 0.01g	65.27 ± 1.13j	40.27 ± 2.33i	32.24 ± 1.08i	59.96 ± 2.37k
RA24	880.78 ± 3.37b	33.92 ± 1.23i	3.24 ± 0.02i	61.57 ± 1.73k	39.78 ± 3.12j	20.57 ± 1.18l	68.66 ± 1.88h
RA25	827.62 ± 4.98l	35.56 ± 1.17h	1.42 ± 0.03t	90.27 ± 1.77b	49.75 ± 1.87c	45.65 ± 1.26d	91.94 ± 1.95a
Grand mean	853.17	33.99	2.93	71.41	43.40	38.86	78.27
CV%	2.245	1.282	0.215	0.545	0.826	0.582	0.518

CV, Coefficient of variation; n = 3; Significant at 1% level; Different letters in each column is differed significantly by Tukey’s HSD test

**Table 2:** Mineral compositions (Macroelements mg g^-1^ DW and microelements μg g^-1^ DW) of 25 red morph amaranth genotypes.

Genotypes	Macroelements (mg g^-1^ DW)			Microelements (μg g^-1^ DW)			
	K	Ca	Mg	Fe	Mn	Cu	Zn
RA1	10.46 ± 0.12gh	24.82 ± 0.17h	29.88 ± 0.99f	904.92 ± 3.65l	332.64 ± 1.27b	29.08 ± 0.03f	980.45 ± 2.12j
RA2	7.46 ± 0 .13l	26.42 ± 0.21f	29.26 ± 0.76g	882.28 ± 4.36m	176.49 ± 1.11m	20.09 ± 0.21l	1020.62 ± 1.88h
RA3	13.86 ± 0.21c	19.22 ± 0.18l	29.26 ± 0.77g	1118.4 ± 4.29f	152.76 ± 1.25n	12.09 ± 0.34o	652.63 ± 1.65t
RA4	7.27 ± 0.16m	27.22 ± 0.15e	28.95 ± 0.46g	1020.57 ± 3.99i	309.23 ± 0.98d	20.06 ± 0.37l	992.12 ± 2.01i
RA5	8.89 ± 0.09j	24.02 ± 0.14h	30.19 ± 0.62e	1035.49 ± 5.08hi	196.48 ± 1.02j	45.12 ± 0.54a	1082.09 ± 3.26f
RA6	11.39 ± 0.17f	32.82 ± 0.72b	31.13 ± 0.81c	881.62 ± 6.23m	264.09 ± 1.17f	18.19 ± 0.61m	720.05 ± 2.54r
RA7	11.63 ± 0.23e	27.15 ± 0.89e	32.05 ± 0,54b	1131.32 ± 4.24e	251.35 ± 1.26g	27.88 ± 0.72g	980.26 ± 1.87j
RA8	10.12 ± 0.17h	31.22 ± 0.76c	30.19 ± 0.55e	1116.91 ± 4.52f	313.76 ± 1.18c	26.07 ± 0.66h	1400.38 ± 1.28c
RA9	7.24 ± 0.14m	16.02 ± 0.73n	30.51 ± 0.62d	1037.67 ± 5.27h	223.31 ± 1.05i	25.54 ± 0.48i	840.65 ± 1.35p
RA10	7.48 ± 0.16l	25.62 ± 0.67g	29.88 ± 0.72f	980.62 ± 6.42j	182.63 ± 1.19l	20.54 ± 0.71l	940.36 ± 1.29m
RA11	12.26 ± 0.08d	19.22 ± 0.85l	29.88 ± 0.78f	748.18 ± 4.36n	174.63 ± 1.22m	42.15 ± 0.54b	741.52 ± 1.18q
RA12	11.28 ± 0.18f	28.22 ± 0.88d	30.19 ± 0.65e	963.08 ± 5.28k	308.75 ± 1.16d	27.43 ± 0.59g	981.43 ± 2.09j
RA13	16.28 ± 0.09a	22.07 ± 0.58j	35.43 ± 0.76a	1525.33 ± 4.81b	356.84 ±1.18a	27.25 ± 0.47g	1473.54 ± 2.02b
RA14	10.75 ±0.16g	24.82 ± 0.87h	24.51 ± 0.77i	1472.26 ± 4.87c	351.39 ± 1.21a	16.03 ± 0.62n	901.11 ± 1.16n
RA15	14.21 ± 0.11b	17.82 ± 0.85m	32.53 ± 0.82b	1727.91 ± 3.88b	333.78 ± 1.06b	23.31 ± 0.74k	1525.92 ± 1.09a
RA16	9.76 ± 0.06i	25.62 ± 0.57g	29.26 ± 0.79g	987.36 ± 3.92j	197.56 ± 0.87j	20.22 ± 0.63l	900.92 ± 1.18o
RA17	6.55 ± 0.08n	24.02 ± 0.65h	29.26 ± 0.86g	983.34 ± 5.38j	242.65 ± 0.88h	26.15 ± 0.68h	950.26 ± 1.26l
RA18	10.62 ± 0.12g	24.02 ± 0.77h	30.51 ± 0.65e	2057.02 ± 6.08a	132.65 ± o.83o	18.06 ± 0.47m	601.37 ± 1.29u
RA19	8.94 ± 0.09j	32.02 ±0.81b	30.51 ± 0.73e	902.63 ± 7.22l	241.15 ± 1.02h	38.05 ± 0.47c	1200.24 ± 1.16e
RA20	10.21 ± 0.08h	21.62 ± 0.44k	29.26 ± 0.83g	980.48 ± 3.65j	244.14 ± 1.18h	26.36 ± 0.38h	1040.21 ± 1.27g
RA21	10.24 ± 0.05h	34.82 ± 0.54a	30.51 ± 0.89e	1055.33 ± 4.65h	192.19 ± 1.26k	24.02 ± 0.37j	841.44 ± 1.19p
RA22	10.69 ± 0.07g	28.02 ± 0.72d	29.88 ± 0.77f	1048.82 ± 325h	291.83 ± 1.15e	32.19 ± 0.53e	1304.92 ± 1.26d
RA23	6.55 ± 0.12n	23.22 ± 0.57i	28.63 ± 0.75h	1375.91 ± 4.87d	245.95 ± 0.98h	34.09 ± 0.28d	950.14 ± 1.14l
RA24	8.65 ± 0.15k	27.22 ± 0.64e	29.88 ± 0.73f	195.12 ± 5.12n	197.38 ± 0.86j	24.15 ± 0.32j	961.21 ± 1.05k
RA25	10.46 ± 0.13gh	16.82 ± 0.66n	28.63 ± 0.65h	1097.61 ± 4.29g	176.21 ± 1.03m	20.12 ± 0.33l	681.38 ± 1.16s
Grand mean	10.13	24.96	30.01	1089.19	243.59	25.77	986.61
CV%	2.021	1.275	1.452	0.384	0.682	0.428	0.238

CV, Coefficient of variation; K, Potassium; Ca. Calcium, Mg, Magnesium; Fe, Iron; Mn, Manganese; Cu, Copper; Zn, Zinc; Different letters in each column is differed significantly by Tukey’s HSD test; n = 3; Significant at 1% level

**Table 3:** Mean performance for antioxidant leaf pigments in 25 vegetable amaranth genotypes.

Genotypes	chlorophyll *a* (mg 100 g^-1^ FW)	Chlorophyll *b* (mg 100 g^-1^ FW)	Chlorophyll *ab* (mg 100 g ^-1^ FW) ±	β-cyanins (μg 100 g^-1^ FW)	β-xanthins (μg 100 g^-1^ FW)	Betalains (μg 100 g^-1^ FW)	Carotenoids (μg g^-1^ FW)
RA1	17.91 ± 0.17r	8.92 ± 0.08r	26.82 ± 0.09m	34.70 ± 0.21i	35.01 ± 0.21g	69.70 ± 0.25i	1585.49 ± 1.24g
RA2	25.73 ± 0.12m	15.08 ± 0.07l	40.80 ± 0.12j	19.98 ± 0.18r	19.47 ± 0.15o	39.44 ± 0.24v	1637.44 ± 1.28e
RA3	53.65 ± 0.11b	27.50 ± 0.05c	81.14 ± 0.03b	53.77 ± 0.11b	53.25 ± 0.24b	107.01 ± 0.28b	1518.68 ± 1.42j
RA4	18.30 ± 0.24q	9.03 ± 0.06r	27.32 ± 0.15m	28.04 ± 0.16m	29.65 ± 0.08j	57.68 ± 0.15o	723.39 ± 2.47x
RA5	15.30 ± 0.17t	9.58 ± 0.08q	24.87 ± 0.11n	27.01 ± 0.09n	26.34 ± 0.24l	53.34 ± 0.18r	1681.72 ± 2.45a
RA6	19.68 ± 0.14p	10.71 ± 0.05o	30.38 ± 0.11l	32.00 ± 0.14k	28.41 ± 0.23k	60.40 ± 0.24n	1052.43 ± 1.68t
RA7	30.56 ± 0.11j	16.08 ± 0.05j	46.63 ± 0.21i	32.93 ± 0.21j	32.90 ± 0.21h	65.82 ± 0.24k	1114.19 ± 2.35s
RA8	45.10 ± 0.07f	26.33 ± 0.07d	71.42 ± 0.25e	56.78 ± 0.07a	58.12 ± 0.07a	114.89 ± 0.16a	1471.17 ± 3.24l
RA9	26.32 ± 0.34l	12.80 ± 0.05m	39.11 ± 0.08k	29.65 ± 0.17l	30.56 ± 0.08i	60.20 ± 0.18n	564.66 ± 3.45y
RA10	20.02 ± 0.24o	9.96 ± 0.04p	29.97 ± 0.23l	33.86 ± 0.18i	34.30 ± 0.15g	68.15 ± 0.75j	1562.15 ± 2.38h
RA11	45.70 ± 0.08e	29.73 ± 0.06a	75.42 ± 0.16d	37.61 ± 0.16g	37.7 0 ± 0.12f	75.30 ± 0.35g	756.89 ± 2.75w
RA12	36.74 ± 0.15h	20.99 ± 0.04g	57.72 ± 0.08f	36.92 ± 0.22h	35.45 ± 0.17g	72.36 ± 0.34h	1460.01 ± 1.68m
RA13	15.39 ± 0.13s	8.79 ± 0.07r	24.17 ± 0.16n	21.87 ± 0.23q	21.38 ± 0.19n	43.24 ± 0.25t	794.39 ± 1.87v
RA14	40.61 ± 0.23g	15.95 ± 0.03k	56.55 ± 0.14f	32.17 ± 0.17k	31.11 ± 0.15i	63.27 ± 0.18l	1540.16 ± 1.52i
RA15	52.99 ± 0.15c	25.84 ± 0.05e	78.82 ± 0.12c	52.09 ± 014c	52.47 ± 0.16b	104.55 ± 0.17c	1363.29 ± 2.45p
RA16	27.72 ± 0.06k	10.80 ± 0.06o	38.51 ± 0.16k	28.59 ± 0.12m	27.77 ± 0.24k	56.35 ± 0.19p	1453.77 ± 3.25n
RA17	23.00 ± 0.16n	8.22 ± 0.08s	31.21 ± 0.14l	21.01 ± 0.16q	20.48 ± 0.18n	41.48 ± 0.24u	1677.26 ± 3.21b
RA18	46.28 ± 0.11d	24.23 ± 0.09f	70.50 ± 0.18e	48.68 ± 0.18d	49.41 ± 0.17c	98.08 ± 0.35d	1652.73 ± 2.34c
RA19	19.38 ± 0.21p	7.32 ± 0.05t	26.69 ± 0.16m	13.96 ± 0.14s	12.57 ± 0.11p0.20	26.52 ± 0.31w	1593.94 ± 2.54f
RA20	27.57 ± 0.32k	11.58 ± 0.03m	39.14 ± 0.12k	26.09 ±0.15o	28.23 ± 0.24k	54.31 ± 0.26q	1642.95 ± 5.21d
RA21	25.60 ± 0.16m	10.00 ± 0.03p	35.59 ± 0.21 l	20.99 ±0.15q	21.45 ± 0.18n	42.43 ± 0.62tu	1494.67 ± 4.25k
RA22	36.61 ± 0.11h	17.60 ± 0.02i	54.20 ± 0.24g	42.78 ± 0.18f	42.41 ± 0.16e	85.18 ± 0.37f	1147.66 ± 3.29r
RA23	26.03 ± 0.29l	15.10 ± 0.05l	41.12 ± 0.28j	24.32 ± 0.16p	25.15 ± 0.14m	49.46 ± 0.28s	1176.91 ± 2.15q
RA24	32.72 ±0.27i	19.81 ± 0.01h	52.52 ± 0.18h	32.85 ± 0.12k	28.39 ± 0.19k	61.23 ± 0.16m	907.87 ± 2.18u
RA25	65.82 ± 0.15a	29.23 ± 0.02b	95.04 ± 0.19a	44.02 ± 0.17e	45.46 ± 0.21d	89.47 ± 0.47e	1384.72 ± 2.48o
Grand mean	31.79	16.05	47.83	33.30	33.09	66.40	1225.94
CV%	3.435	2.352	2.186	2.682	3.524	3.338	4.287

CV, Coefficient of variation; n = 3; Significant at 1% level; Different letters in each column is differed significantly by Tukey’s HSD test

**Table 4:** Mean performance for β-carotene, vitamin C, TPC, TFC, TAC (DPPH) and TAC (ABTS^+^) of 25 vegetable amaranth genotypes.

Genotypes	β-carotene (μg g^-1^ FW)	Vitamin C (μg g^-1^ FW)	TPC (GAE μg g^-1^ DW)	TFC (RE μg g^-1^ DW)	TAC (DPPH) (TEAC μg g^-1^ DW)	TAC (ABTS^+^) (TEAC μg g^-1^ DW)
RA1	1214.12 ± 2.14g	621.38 ± 1.82p	205.24 ± 0.15g	35.19 ± 0.15s	11.17 ± 0.22g	21.86 ± 0.34i
RA2	1249.82 ± 1.17f	1052.40 ± 1.09h	169.12 ± 0.24j	75.94 ± 0.24m	15.60 ± 0.12f	30.53 ± 0.35g
RA3	1099.81 ± 2.38n	1791.62 ± 2.14c	257.33 ± 0.35b	127.89 ± 0.33e	26.56 ± 0.14c	51.97 ± 0.36c
RA4	559.29 ± 2.46v	929.08 ± 2.07k	108.13 ± 0.46o	78.44 ± 0.27l	17.80 ± 0.22e	34.83 ± 0.47f
RA5	1524.41 ± 2.78a	1990.58 ± 2.26a	232.63 ± 0.44d	164.49 ± 0.18b	30.90 ± 0.35a	60.47 ± 0.38a
RA6	807.99 ± 2.19r	1727.24 ± 1.84d	126.04 ± 0.25n	57.80 ± 0.09q	17.50 ± 0.31e	34.24 ± 0.25f
RA7	851.91 ± 2.34q	760.27 ± 1.46o	150.75 ± 0.92l	47.23 ± 0.22r	18.76 ± 0.22e	36.71 ± 0.32f
RA8	1162.92 ± 3.47j	1221.12 ± 1.95g	199.42 ± 0.88h	141.81 ± 0.24d	28.81 ± 0.24b	56.38 ± 0.33b
RA9	434.02 ± 3.62w	1360.32 ± 2.08f	127.71 ± 0.75n	86.31 ± 0.26k	17.50 ± 0.18e	34.24 ± 0.13f
RA10	1194.85 ± 2.59h	806.20 ± 2.29n	160.34 ± 0.38k	68.33 ± 0.25o	11.46 ± 0.34g	22.42 ± 0.27i
RA11	584.00 ± 2.06u	621.14 ± 2.05p	177.42 ± 0.44i	119.83 ± 0.35g	23.86 ± 0.21d	46.69 ± 0.43d
RA12	1124.02 ± 3.79l	436.00 ± 1.72r	150.14 ± 0.62l	93.69 ± 0.34j	11.19 ± 0.22g	21.89 ± 0.26i
RA13	608.72 ± 3.58t	805.51 ± 1.78n	96.21 ± 0.52q	71.46 ± 0.17n	14.52 ± 0.34f	28.41 ± 0.27g
RA14	1172.80 ± 2.75i	810.52 ± 1.64m	140.16 ± 0.58m	62.23 ± 0.22p	17.10 ± 0.33ef	33.46 ± 0.22fg
RA15	1285.83 ± 2.16d	1044.19 ± 1.57i	260.84 ± 0.47a	171.26 ± 0.27a	27.49 ± 0.35c	53.79 ± 0.12c
RA16	1113.19 ± 2.26m	867.14 ± 1.02l	150.23 ± 0.63l	58.60 ± 0.29q	15.03 ± 0.38f	29.41 ± 0.18g
RA17	1296.83 ± 2.84c	374.33 ± 0.98s	172.59 ± 0.42j	114.77 ± 0.28h	14.72 ± 0.42f	28.80 ± 0.24g
RA18	1431.03 ±2.94b	1620.90 ± 3.74e	223.43 ± 0.38e	151.48 ± 0.19c	31.53 ± 0.16a	61.70 ± 0.26a
RA19	1215.98 ± 2.29g	1853.01 ± 2.24b	205.41 ± 0.29g	98.79 ± 0.42i	22.39 ± 0.35d	43.81 ± 0.22e
RA20	1247.93 ± 1.83f	313.12 ± 2.18t	160.09 ± 0.42k	121.22 ± 0.35f	12.43 ± 0.37g	24.32 ± 0.27h
RA21	1145.05 ± 2.53k	436.43 ± 2.04r	102.40 ± 0.26p	126.33 ± 0.17e	15.60 ± 0.26f	30.53 ± 0.62g
RA22	875.84 ± 2.44p	103.53 ± 1.32u	179.15 ± 022i	98.50 ± 0.44i	29.83 ± 0.28ab	58.37 ± 0.28ab
RA23	908.73 ± 3.52o	806.92 ± 2.07n	102.62 ±0.51p	119.18 ± 0.33g	17.42 ± 0.17e	34.09 ± 0.27f
RA24	698.82 ± 3.37s	497.70 ± 0.88q	205.78 ± 0.48f	96.03 ± 0.28i	18.53 ± 0.19e	36.26 ± 0.32f
RA25	1271.71 ± 3.25e	1029.15 ± 1.45j	250.08 ± 0.77c	165.80 ± 0.27b	31.70 ± 0.24a	62.03 ± 0.29a
Grand mean	1043.18	955.19	172.53	102.10	19.97	39.09
CV%	3.978	2.257	3.615	1.254	1.026	0.865

CV, Coefficient of variation; TAC, Total antioxidant capacity; TPC, Total polyphenol content; TFC, Total flavonoid content; n = 3; Significant at 1% level; Different letters in each column is differed significantly by Tukey’s HSD test

### Proximate compositions

Proximate compositions of red morph amaranth are presented in Table 1. The genotype RA11 exhibited the highest moisture content (884.73 g kg^-1^ FW), while the genotype RA3 and RA18 had the lowest moisture content (814.64 and 814.83 g kg^-1^ FW). The moisture content ranged from 814.64 to 884.73 g kg^-1^ FW. Red morph amaranth leaves exhibited noticeable variations in protein content. The genotype RA3 had the highest protein content (62.26 g kg^-1^) followed by RA11 and RA15, whereas the genotype RA6 exhibited the lowest protein content (11.38 g kg^-1^). For protein content, ten genotypes performed better over their mean value. Among them, eight genotypes RA3, RA8, RA11, RA15, RA5, RA9, RA18, and RA19 showed higher protein content as leafy vegetables. The highest fat content was observed in the genotype RA7 (4.35 g kg^-1^ FW) against the lowest content recorded for the genotype RA25 (1.42 g kg-1 FW) with an average of 2.93 g kg^-1^ FW.

The highest carbohydrates content were noted in the genotype RA16 and RA6 (98.54 and 97.51 g kg^-1^ FW) followed by RA1, RA25, and RA20, while the lowest carbohydrates content was observed in RA19 (15.48 g kg^-1^ FW) with an average of 71.41 g kg^-1^ FW. The genotype RA18 and RA3 had the highest energy (56.07 and 55.33 Kcal 100 g^-1^ FW) followed by RA3, RA8, RA15, and RA25, while the lowest energy was obtained from the genotype RA19 (26.95 Kcal 100g^-1^ FW) with an average of 43.40 Kcal 100 g^-1^ FW. Ash content was the highest in the genotype RA3 (56.55 g kg^-1^ FW) followed by RA8, RA5, RA19, RA15, and RA18, while the lowest ash content was noted in RA24 (20.57 g kg^-1^ FW) with an average of 38.86 g kg^-1^ FW. Dietary fiber content exhibited remarkable variations in 25 red morph amaranth studied. The dietary fiber content was the highest in RA25 and RA2 (91.94 and 91.66 μg g^-1^ FW) followed by RA2, RA4, RA22, RA21, RA9, RA1, RA16, RA8, and RA20, while RA23 exhibited the lowest dietary fiber content (59.96 μg g^-1^ FW) with an average of 78.27 μg g^-1^ FW.

### Mineral compositions

Mineral compositions of red morph amaranth are presented in Table 2. In the present investigation, K content ranged from 6.55 mg g^-1^ to 16.28 mg g^-1^ DW. The genotypes RA13, RA15, RA3, RA11, and exhibited high K content, while genotype RA17 and RA23 showed the lowest K content with an average of 10.13 mg g^-1^ DW. Thirteen genotypes performed much better than their average performance of K content. Calcium content ranged from 16.02–34.82 mg g^-1^ DW.

The genotypes RA21, RA6, RA19, RA8, RA12, and RA22 showed high Ca content, while the genotype RA9 and RA25 had the lowest Ca content with an average Ca content of 24.96 mg g^-1^ DW. Twelve genotypes had better Ca content over their corresponding mean. Mg content was the highest in RA13 and the lowest in RA14, with an average of 30.01 mg g^-1^ DW. The genotype RA13, RA15, RA7, RA6, RA9, RA18, RA19, RA21, RA5, RA8, and RA12 showed higher Mg content. In this study, the genotypes did not show considerable variations in terms of Mg content (24.51 to 35.43 mg g^-1^ DW).

The significant and remarkable variations were detected for iron content (195.12 μg g^-1^ DW in RA24 to 2057.02 μg g^-1^ DW in RA18). The genotypes RA18, RA15, RA13, RA14, and RA23 exhibited the highest iron content. Conversely, the genotype RA24 showed the lowest iron content, with an average value of 1089.19 μg g^-1^ DW. Nine genotypes had higher iron content over their average performance. In this study, the manganese content ranged between 132.65 μg g^-1^ DW and 356.84 μg g^-1^ DW, with an average of 243.59 μg g^-1^ DW. The genotype RA13, RA14, RA15, RA13, RA1, RA8, RA4, and RA12 had high manganese content, however, the genotype RA18 showed the lowest manganese content (132.65 μg g^-1^ DW). The copper content had significant and notable variations in the studied genotypes (12.09–45.12 μg g^-1^ DW). The highest copper content was noted in RA5 (45.12 μg g^-1^ DW), followed by RA11, and RA19. Twelve genotypes exhibited higher Cu content over their corresponding grand mean. The genotypes differed significantly and remarkably in zinc content (601.37 μg g^-1^ DW (RA18) to 1525.92 μg g^-1^ DW (RA15). Eight genotypes showed higher zinc content over their mean performance (986.61 μg g^-1^ DW).

### Antioxidants leaf pigments

Antioxidant leaf pigments of red morph amaranth are presented in Table 3. Chlorophyll *a* content (15.30 to 65.82 mg 100 g^-1^) exhibited prominent variations among genotypes. The highest chlorophyll *a* content (65.82 mg 100 g^-1^) was observed in the genotype RA25, while the genotype RA5 showed the lowest chlorophyll *a* content (15.30 mg 100 g^-1^).

The genotypes RA3 and RA15 had high chlorophyll *a* content. Ten genotypes exhibited higher chlorophyll *a* content over their resultant grand mean. Like chlorophyll *a*, significant and marked differences were observed in chlorophyll *b* content (7.32 to 29.73 mg 100 g^-1^) in 25 red morphs amaranth genotypes. The highest chlorophyll *b* content (29.73 mg 100 g^-1^) was recorded in the genotype RA11 followed by RA25, RA3, RA8, RA15, and RA18. In contrast, the genotype RA19 exhibited the lowest chlorophyll *b* content (7.32 mg 100 g^-1^). Chlorophyll *ab* showed significant and remarkable variation (24.17 to 95. 04 mg 100 g^-1^). The genotype RA3, RA69, RA15, RA11, RA8, and RA18 exhibited high chlorophyll *ab* content, whereas, the lowest chlorophyll *ab* content was recorded in RA13 (24.17 mg 100 g^-1^). Ten genotypes had higher chlorophyll *ab* content over their mean value. β-cyanins ranged from 13.96 to 56.78 μg 100 g^-1^ with an average value of 33.30 μg 100 g^-1^. The genotype RA8 exhibited the highest β-cyanins content (56.78 μg 100 g^-1^) followed by RA3, RA15, RA18, and RA25. Conversely, the genotype RA19 showed the lowest β-cyanins content (13.96 μg 100 g^-1^). Among genotypes, significant and remarkable variations were observed in β-xanthins content, with a range of 12.57 to 58.12 μg 100 g^-1^. β-xanthins content was the highest in RA8 (58.12 μg g^-1^) and higher in RA3, RA15, RA18, and RA25. On the other hand, the genotype RA19 showed the lowest β-xanthins content (12.57 μg 100 g^-1^). Ten genotypes showed better performance over their grand mean. Betalains varied significantly and markedly and ranged from 26.52 to 114.89 μg 100 g^-1^. The genotype RA8 had the highest betalains content (114.89 μg 100 g^-1^), and genotype RA3, RA15, RA18, and RA25 had higher betalains content. Whereas, the genotype RA19 had the lowest betalains content (26.52 μg 100 g^-1^). Nine genotypes showed better performance over the grand mean. Total carotenoids content ranged from 564.66 μg g^-1^ in RA9 to 1677.26 μg g^-1^ in RA17. The genotype RA18, RA20, RA5, RA2, RA1, RA3, RA49, RA10, RA58, RA14, and RA19 had high total carotenoids. Fourteen genotypes had better performance over their mean value.

### Antioxidant phytochemicals and antioxidant activity

Vitamins, TAC, TFC, and TPC of red morph amaranth are presented in Table 4. β-Carotene content ranged from 559.29 μg g^-1^ in RA4 to 1524.41 μg g^-1^ in RA5. The genotype RA5 exhibited the highest β-carotene content (1524.41μg g^-1^) and the genotype RA18, RA25, RA15, RA20, and RA2 demonstrated high β-carotene content. Sixteen genotypes performed better than their grand mean. Vitamin C content ranged from 103.53 μg g^-1^ in RA22 to 1990.58 μg g^-1^ in RA5 with an average of 955.19 μg g^-1^. The genotype RA3, RA19, RA6, and RA18 exhibited high vitamin C content. Ten genotypes performed better over their respective grand mean. Total polyphenol content (TPC) ranged from 96.21 GAE μg g^-1^ DW (RA13) to 260.84 GAE μg g^-1^ DW (RA15) with an average TPC content of 172.53 GAE μg g^-1^ DW. The genotype RA15 had the highest TPC. The genotype RA25, RA3, RA5, and RA18 had higher TPC values. Eleven genotypes had a higher performance of TPC over their respective grand mean. TFC exhibited much noticeable variation in terms of genotypes, which ranged from 35.19 RE μg g^-1^ DW in the genotype RA1 to 171.26 RE μg g^-1^ DW in the genotype RA15. The average mean of TFC was 102.10 RE μg g^-1^ DW. The genotype RA15 exhibited the highest TFC showing the following order: RA15 ˃ RA25 ˃ RA5 ˃ RA18. Eleven genotypes exhibited better performance over their respective grand mean. TAC (DPPH) ranged from 11.17 TEAC μg g^-1^ DW (RA1) to 31.70 TEAC μg g^-1^ DW (RA25). The higher TAC (DPPH) was recorded in the genotype RA18, RA5, RA22, RA8, RA15, and RA3. In contrast, the genotype RA1, RA10, and RA20 had the lowest TAC (DPPH) with an average of 19.25 TEAC μg g^-1^ DW. Nine genotypes had better performance over their respective grand mean. TAC (ABTS^+^) ranged from 21.86 TEAC μg g^-1^ DW (RA1) to 62.03 TEAC μg g^-1^ DW (RA25). The genotype RA25 had the highest TAC (ABTS^+^) which was statistically similar to the genotype RA18, RA5, and RA22. The higher TAC (ABTS^+^) was noticed in the genotypes, RA8, RA15, and RA3. In contrast, TAC (ABTS^+^) was the lowest in RA1 (21.86 μg g^-1^ DW), RA12 (21.89 μg g^-1^ DW), and RA10 (22.42 μg g^-1^ DW) with an average of 39.09 TEAC μg g^-1^ DW. Nine genotypes showed much better performance over their respective mean betalains.

### Correlation coefficient analysis

Correlation of bioactive compounds of red morph amaranth is presented in Table 5. The analysis of correlation coefficients presented in Table 5 exhibited exciting results. The chlorophyll *ab*, chlorophyll *b*, and chlorophyll *b* exhibited positive and significant associations among each of them and with β-cyanins, β-xanthins, betalains, TAC (ABTS^+^), TFC, TPC, and TAC (DPPH). Carotenoids and β-carotene exhibited a significant negative association with all leaf pigments, whereas, these two traits had a significant positive relationship with TAC (ABTS^+^), TFC, TPC, and TAC (DPPH). Carotenoids and β-carotene were positively associated with each other. Conversely ascorbic acid exhibited insignificant interrelationships with all the traits. TAC (ABTS^+^), TFC, TPC, and TAC (DPPH) exhibited substantial positive association among each other, all leaf pigments, and vitamins.

**Table 5:** Correlation coefficient for antioxidant leaf pigments, β-carotene, vitamin C, TPC, TFC, TAC (DPPH) and TAC (ABTS+) in 25 vegetable amaranth genotypes.

Traits	Chl *b* (mg 100 g^-1^ FW)	Chl *ab* (mg 100 g^-1^ FW)	β-cyanins (μg 100 g^-1^ FW)	β- xanthin (μg 100 g^-1^ FW)	Betalains (μg 100 g^-1^ FW)	Catonenoirds (μg g^-1^ FW)	β-carotene (μg g^-1^ FW)	Vitamin C (μg g^-1^ FW)	TPC (GAE μg g^-1^ DW)	TFC (RE μg g^-1^ DW)	TAC (TEAC μg g^-1^ DW)	TAC (ABTS^+^) (TEAC μg g^-1^ DW)
Chlorophyll *a* (mg100 g^-1^ FW)	0.93**	0.88**	0.73**	0.74**	0.72**	-0.58**	-0.48**	-0.001	0.48**	0.48**	0.65**	0.67**
Chlorophyll *b* (mg 100 g^-1^ FW)		0.89**	0.68**	0.68**	0.69**	-0.57**	-0.54**	-0.011	0.47**	0.55**	0.63**	0.61**
Chlorophyll *ab* (mg 100 g^-1^ FW)			0.76**	0.76**	0.66**	-0.66**	-0.56**	-0.007	0.52**	0.54**	0.61**	0.68**
β-cyanins (μg 100 g^-1^ FW)				0.87**	0.88**	-0.76**	-0.58**	-0.10	0.58**	0.58**	0.71**	0.74**
β-xanthins (μg 100 g^-1^ FW)					0.92**	-0.69**	-0.59**	-0.12	0.62**	0.56**	0.64**	0.75**
Betalains (μg 100 g^-1^ FW)						-0.72**	-0.63**	-0.11	0.64**	0.62**	0.75**	0.79**
Catonenoirds (μg g^-1^ FW)							0.88**	-0.18	0.58**	0.55**	0.73**	0.88**
β-carotene (μg g^-1^ FW)								-0.17	0.39*	0.48**	0.64**	0.68**
Vitamin C (μg g^-1^ FW)									0.05	0.02	0.06	0.08
TPC (GAE μg g^-^1 DW)										0.77**	0.68**	0.98**
TFC (RE μg g^-1^ DW)											0.79**	0.86**
TAC (DPPH) (TEAC μg g^-1^ DW)												0.99**

Chl *a*, Chlorophyll *a;* Chl *ab*, Chlorophyll *ab;* TAC, Total antioxidant capacity; TPC, Total polyphenol content; TFC, Total flavonoid content

* Significant at 5% level

** Significant at 1% level.

## Discussion

Color, flavor, and taste predominantly influenced the acceptability of foods products. Considering the safety, nutritional, and aesthetic aspects of food, the demand for natural pigments have been increased in consumers day by day. Red morph amaranth was a unique and inexpensive source of color pigments such as β-cyanins, β-xanthins, betalains, anthocyanin, amaranthine, carotenoids, and chlorophylls that have potential free radical detoxifying ability and act as potent antioxidants [1,3]. The active ingredients of these pigments have significant contributions to human health as they provide protection against lung and skin cancers, cardiovascular and inflammatory disease [4]. Red morph amaranth also had abundant natural antioxidant phytochemicals such as flavonoids, β-carotene, phenolics, and vitamin C along with protein, dietary fiber, and minerals. These natural antioxidants protect cancer, emphysema, cardiovascular diseases, atherosclerosis, diabetes, retinopathy, osteoporesis, neurodegenerative diseases, arthritis, cataracts, inflammation, and prevent aging [23–25]. It is a widely distributed leafy vegetable in Bangladesh, Asia, Africa and South America with great variability and phenotypic plasticity [22]. In Bangladesh, red morph amaranth was grown year-round and in the hot summer, a gap period of foliage vegetables [11–12]. Its attractive leaf color, nutritional value, and taste make it popular in the rest of the continent and elsewhere. Its production and consumption have been remarkably increased due to the presence of excellent nutritional and natural antioxidants such as antioxidant leaf pigments, vitamins, phenolics, and flavonoids. So, red morph amaranth could significantly contribute to local, regional and international nutritional and antioxidants security challenges by reducing the hidden hunger and attaining nutritional and antioxidant sufficiency in the world. In this study, we comprehensively evaluated 25 red morph amaranth genotypes in terms of proximate and mineral compositions, antioxidant pigments, phytochemicals, and antioxidant activity and their variability. Our results demonstrated that red morph amaranth had abundant natural pigments, phytochemicals with high antioxidant activity along with nutritional components. However, the components varied significantly in terms of genotypes.

One of the interesting findings of our study was that we obtained abundant antioxidant pigments and phytochemicals with high antioxidant activity along with nutritional components in the red morph amaranth genotypes which could contribute to local, regional, and international nutritional and antioxidant sufficiency by reducing the hidden hunger and attaining antioxidant sufficiency in the world. We found remarkable chlorophyll *a* (31.79 mg 100 g^-1^), chlorophyll *b* (16.05 mg 100 g^-1^) and chlorophyll *ab* (47.83 mg 100 g^-1^) content in the red morphs amaranth, whereas, Khanam and Oba [32] observed comparatively lower chlorophyll content in red morphs amaranth. We observed remarkable β-cyanins (56.78 μg 100 g^-1^), β-xanthins (58.12 μg 100 g^-1^), betalains (114.89 μg 100 g^-1^), and total carotenoids (1677.26 μg g^-1^) in the red morphs amaranth, similarly, Khanam and Oba [32] observed more or less similar trend in β-cyanins, β-xanthins, betalains, and total carotenoids content of red morphs amaranth. Regarding phytochemicals, we found remarkable β-carotene (1524.41 μg g^-1^), vitamin C (1990.58 μg g^-1^) in the red morphs amaranth, which exhibited comparatively higher values in terms of our previous studies in *A*. *tricolor* [12]. TPC (260.84 GAE μg g^-1^ DW) obtained in this study were also higher than the results of Khanam and Oba [32] in *A*. *tricolor*. TFC (171.26 RE μg g^-1^ DW), TAC (DPPH) (31.70 TEAC μg g^-1^ DW), and TAC (ABTS^+^) (62.03 TEAC μg g^-1^ DW) obtained in this study were more or less similar to the results of Khanam et al. [33] in *A*. *tricolor*. The genotypes RA25 had the highest TAC (DPPH, ABTS+), chlorophylls, high betalains, total carotenoids, β-carotene, phenolics, and flavonoids. The genotype RA18 had high TAC (DPPH, ABTS+), chlorophylls, betalains, total carotenoids, β-carotene, phenolics, and flavonoids. The genotype RA15 exhibited high TAC (DPPH, ABTS+), chlorophylls, betalains, total carotenoids, β-carotene, and the highest phenolics and flavonoids. The genotype RA5 and RA3 had high TAC (DPPH, ABTS+), chlorophylls, betalains, total carotenoids, moderate β-carotene, phenolics, and flavonoids. These five genotypes could be used as antioxidant profile enriched high-yielding varieties. We can conclude that red amaranth has abundant phenolics, pigments, flavonoids, vitamins, and antioxidant that offered enormous prospects for nourishing the vitamin and antioxidant scarce people.

As lower moisture content was desirable to confirm the higher dry matter, six genotypes such as RA3, RA18, RA15, RA8, RA5, and RA25 showed 18–19% dry matter might be selected for dry matter. The moisture content of red morph amaranth leaves directly related to the maturity of the plant. Similar results were reported by Sun et al. on sweet potato leaves [34]. Eight genotypes RA3, RA8, RA11, RA15, RA5, RA9, RA18, and RA19 showed higher protein content as leafy vegetables. Vegetarian and poor people of the low-income countries mainly depend on red amaranth for their protein source. So, red morph amaranth might be an excellent source of protein for vegetarian and poor people. The protein content of red morph amaranth (33.99 g kg^-1^) was much higher as compared to *A*. *tricolor* (1.26%) in our earlier study [2]. The fat content in the present study agreed to the results of Sun et al. [34] in sweet potato leaves. They mentioned that fat involved in the insulation of body organs and maintenance of body temperature and cell function. Fats have abundant omega-3 and omega-6 fatty acids. Fats play a significant role in absorption, digestion, and transport of fat-soluble vitamins A, D, E, and K. Dietary fiber has a significant role in palatability, digestibility, and remedy of constipation [14]. From the results, we observed that red morph amaranth leaves have abundant carbohydrates, protein, moisture, and dietary fiber. As red morph amaranth had low energy content, this may not impact significantly on energy contribution to the human body as low amounts of this vegetable consumed in a day. Like other leafy vegetables, the low carbohydrate content of red morph amaranth may not have a significant impact on carbohydrate contribution to the human body considering the low amount of vegetable uptake per day and a very high daily requirement for the human body.

Amaranth had higher mineral contents than commonly consumed leafy vegetables, such as spinach, lettuce, and kale [35]. In our present study, we found remarkable K (10.13 mg g^-1^), Ca (24.96 mg g^-1^), and Mg (30.01 mg g^-1^) in the red morph amaranth, albeit we estimated in dry weight basis. Jimenez-Aguiar and Grusak [35] noted abundant K, Ca, and Mg in different amaranths including red amaranth. They also found amaranth K, Ca, and Mg was much higher than spinach, spider flower, kale, and black nightshade. Zinc and iron content of red morph amaranth is higher than the cassava leaves [36] and beach pea [37]. In this study, we found remarkable Fe (1089.19 μg g^-1^), Mn (243.59 μg g^-1^), Cu (25.77 μg g^-1^), and Zn (996.61 μg g^-1^) in red morph amaranth, although we estimated in dry weight basis. Similarly, Jimenez-Aguiar and Grusak [35] noted abundant Fe, Mn, Cu, and Zn in different amaranths including red amaranth. They also found amaranth Fe, Mn, Cu, and Zn were much higher than spinach, spider flower, kale, and black nightshade. The U.S. Department of Agriculture’s National Nutrient Database for Standard Reference [35] lists a serving size of spinach as 30 g fresh weight FW (1 cup). As red amaranth has higher mineral concentrations than spinach so, a serving size of leaves of 30 g FW is enough for nutritional sufficiency.

The chlorophyll *ab*, chlorophyll *b*, and chlorophyll *b* exhibited positive and significant associations among each of them and with β-cyanins, β-xanthins, betalains, TAC (ABTS^+^), TFC, TPC, and TAC (DPPH). It revealed that increment of one leaf pigment straightly associated with an increase of another leaf pigment. The significant positive correlation of all leaf pigments with TAC (ABTS^+^), TFC, TPC, and TAC (DPPH) signifies that all the leaf pigments had strong antioxidant activity. Significant positive association of carotenoids and β-carotene with TAC (ABTS^+^). TFC, TFC, TPC, and TAC (DPPH) suggested that carotenoids and β-carotene had strong antioxidant activity. Ascorbic acid exhibited insignificant interrelationships with all the traits indicating negligible contribution in the antioxidant potentiality of red morphs amaranth. Jimenez-Aguilar and Grusak [35] reported similar findings for ascorbic acid in amaranth. A similar trend of the insignificant association was also observed by Shukla et al. [38] in their earlier work in amaranth. TAC (ABTS^+^), TFC, TPC, and TAC (DPPH) exhibited substantial positive association among each other, all leaf pigments, and vitamins representing the involvement of these phytochemicals in antioxidant activity. In the present investigation, it revealed that phenolics, pigments, flavonoids, and vitamins contribute significantly in the antioxidant potentiality of red morphs amaranth.

## Conclusions

Red morphs amaranth leaves have abundant antioxidant pigments and phytochemicals such as β-carotene, chlorophyll, vitamin C, β-cyanins, carotenoids, β-xanthins, TAC, betalains, TPC, and TFC. It also has abundant protein, dietary fiber, and minerals such as Ca, K, Mg, Fe, Cu, Zn, and Cu compared to leafy vegetables. Correlation study revealed that natural antioxidant pigments and phytochemicals had strong antioxidant capacity. It could be a potential leafy vegetable as a source of natural antioxidant pigments and phytochemicals having strong antioxidant activity along with nutritional components in our daily diet to combat with the hidden hunger and attaining nutritional and antioxidant sufficiency. Hence, red morphs amaranth with rice, wheat, and maize in our daily diet could contribute to attaining nutritional and antioxidant sufficiency.
